# Genome-wide subgenome-resolved analysis validates chromosome 4 differentiation and prioritizes introgressed *Coffea arabica* accessions

**DOI:** 10.3389/fpls.2026.1925916

**Published:** 2026-08-31

**Authors:** Ezekiel Ahn, Sunchung Park, Insuck Baek, Moon S. Kim, Lyndel W. Meinhardt

**Affiliations:** 1 Sustainable Perennial Crops Laboratory, Agricultural Research Service, United States Department of Agriculture, Beltsville, MD, United States; 2 Environmental Microbial and Food Safety Laboratory, Agricultural Research Service, United States Department of Agriculture, Beltsville, MD, United States

**Keywords:** allopolyploidy, chromosome 4, *Coffea arabica*, introgression, population genomics, sensitivity analysis, subgenome filtering

## Abstract

Chromosome 4 introgression in Timor hybrid-derived *Coffea arabica* is established, but the robustness of accession prioritization and the relative strength of cultivated–introgressed differentiation across the canephora-derived (sgC) and eugenioides-derived (sgE) subgenomes remained unclear under explicit subgenome filtering. We reanalyzed public genomic resources from 44 coffee accessions using strict contig-level subgenome filtering, Arabica-only population-structure analysis, SNP-panel sensitivity testing, genome-wide differentiation scans, permutation testing, and direct sequence alignment. Population structure and accession rankings were stable across marker densities and random seeds, and the same six introgressed references were retained throughout. Chromosome 4 ranked first in both subgenomes, with a strong sgC signal and a markedly weaker sgE signal; independent baseline-panel permutation tests supported both chromosome 4-associated signals. Direct alignment supported correspondence to the expected chromosome 4 pseudomolecules while showing incomplete source coverage and unresolved exact boundaries. Alignment-supported blocks contained 88 sgC and 62 sgE provisional defense-, signaling-, and regulatory-associated annotations. These results provide a genome-wide, quantitatively validated framework for prioritizing introgressed germplasm and candidate chromosome 4 regions for phenotype-linked coffee research without implying equivalent introgression, exact liftover, or causal resistance genes.

## 1 Introduction

The genus *Coffea* includes diverse diploid species, whereas cultivated coffee production is dominated by diploid *C. canephora* and allotetraploid *C. arabica*. Arabica originated through hybridization between ancestors related to *C. canephora* and *C. eugenioides*, leaving modern accessions with a canephora-derived subgenome (sgC) and an eugenioides-derived subgenome (sgE) (Merot-L’anthoene et al., 2019; Scalabrin et al., 2020; Salojärvi et al., 2024). Its recent origin, dispersal through a small number of cultivated lineages, and subsequent bottlenecks produced an unusually narrow genetic base (Anthony et al., 2002; Gaut et al., 2018), which constrains improvement under changing climates and persistent disease pressure (Ngure and Watanabe, 2024).

Introgression has therefore been central to Arabica improvement. Híbrido de Timor and related materials, derived from *C. arabica*–*C. canephora* hybridization, have contributed disease-resistance-associated diversity to Catimor-, Sarchimor-, and other breeding backgrounds (Lashermes et al., 2000; Herrera et al., 2002; Clarindo et al., 2013; Van der Vossen et al., 2015). Such germplasm can also carry quality or agronomic trade-offs, making accession identity and genomic context important for downstream breeding decisions (Bertrand et al., 2003; Lashermes et al., 2011). More broadly, studies of perennial domestication and crop-wild gene flow show that introgression can contribute adaptive variation while leaving strongly nonuniform genomic signatures (Hufford et al., 2013; Gaut et al., 2015).

Salojärvi et al. (2024) established a shared Timor-derived chromosome 4 introgression region, primarily in sgC, and connected that region with resistance-associated gene space. Accurate subgenome assignment is essential in allopolyploids because homoeologous exchange, structural variation, and analytical misassignment can distort subgenome-specific evolutionary inference (Deb et al., 2023; Scalabrin et al., 2024; Session, 2024). The present study does not claim *de novo* discovery of that signal. Instead, it addresses four complementary questions that were not quantified in the source analysis: whether chromosome 4 remains the strongest signal after strict contig-level subgenome filtering; whether the result is stable across SNP panel sizes and random seeds; which introgressed accessions are prioritized reproducibly; and whether provisional annotation context is supported by direct sequence alignment rather than proportional coordinate scaling alone. The weaker sgE chromosome 4 signal reported below is described as matched group differentiation because it is observed in the same cultivated-introgressed accession contrast; this descriptive term does not imply physical linkage, equivalent alien introgression, or a shared causal mechanism.

**Table 1** contrasts the previously reported biological discovery with the quantitative validation framework and reusable outputs. The workflow explicitly separates sgC- and sgE-labelled pseudomolecules, uses Arabica-only PCA for within-Arabica inference, evaluates sampling and reference-panel sensitivity, performs an unbiased genome-wide scan, and restricts candidate annotations to direct alignment-supported blocks.

**Table 1:** Relationship between the previously reported chromosome 4 introgression result and the quantitative validation framework.

Component	Previously reported study (Salojärvi et al., 2024)	Quantitative validation framework
Chromosome 4 result	Timor-derived *C. canephora* introgression was identified primarily in sgC.	An unbiased genome-wide scan quantitatively validates chromosome 4 as the top pseudomolecule after strict subgenome filtering.
Subgenome interpretation	Direct introgression was interpreted primarily in sgC.	sgC retains a strong signal; sgE shows a weaker chromosome 4-associated differentiation signal in the same accession contrast, without a claim of equivalent introgression.
Robustness analysis	Population-genomic and expression evidence established the biological region.	Four SNP panel sizes, five seeds, all-eligible PCA, Arabica-only sensitivity, all-eligible genome-wide scanning, and 500 label permutations of the strict-filter 12,000-SNP baseline quantify robustness.
Accession-level output	Timor-derived materials were classified as introgressed or hybrid accessions.	A stable top-six accession ranking is recovered in every sensitivity condition, with geographic and pedigree context summarized in **Supplementary Table S1**.
Coordinate and annotation output	Resistance-associated gene space was reported in the chromosome 4 region.	Direct sequence alignment validates chromosome-level correspondence and defines alignment-supported provisional candidate annotations; exact boundaries and causality are not inferred.

Accordingly, we reanalyzed the public 44-accession panel to (i) quantify strict subgenome-specific population structure, (ii) test the stability of chromosome-level differentiation and accession ranking, (iii) compare full-panel and Arabica-only ordinations, and (iv) replace proportional annotation projection with direct sequence-alignment-supported annotation summaries.

## 2 Materials and methods

### 2.1 Public genomic resources and study design

The analysis used public resources associated with Salojärvi et al. (2024): accession metadata and two Arabica variant-call files distributed as sgC and sgE callsets. The panel comprised 39 C*. arabica* accessions classified as cultivated, introgressed, or wild, together with three *C. canephora* and two *C. eugenioides* reference accessions. ET-39 sgC and sgE genome assemblies and GFF3 annotations were obtained from the public *Coffea* assembly collection, and assembly metadata were retrieved through NCBI Datasets. No new plant material, sequencing, or phenotype data were generated.

### 2.2 Metadata harmonization, accession context, and strict subgenome filtering

Accession identifiers were reconciled by case- and punctuation-insensitive normalization and conservative removal of source-specific suffixes. Distributed metadata supplied accession name, country or source, donor institute, pedigree notes, and varietal classification. Biological context for the six prioritized introgressed references was compiled from those metadata and relevant coffee-breeding literature; it is presented as contextual information only because no disease, quality, or agronomic phenotypes were measured in this study (**Supplementary Table S1**).

Both public VCFs were called against a combined Arabica reference and contained sgC-labelled pseudomolecules, sgE-labelled pseudomolecules, and unassigned scaffolds. The workflow therefore restricted the sgC analysis to the 11 contigs explicitly labelled “_sg_C_” and the sgE analysis to the 11 contigs explicitly labelled “_sg_E_” before any PCA, ranking, or differentiation analysis. Opposite-subgenome pseudomolecules and unassigned scaffolds were excluded. The audit retained 23,225,336 sgC-labelled and 25,062,481 sgE-labelled records and excluded 5,768,784 and 6,991,157 opposite-subgenome records, respectively (**Supplementary Table S2**).

### 2.3 Variant filtering and SNP-panel sensitivity

Analyses were restricted to biallelic SNPs with site call rate at least 0.80 and minor allele frequency at least 0.05, yielding 3,601,265 eligible sgC variants and 5,317,075 eligible sgE variants. A strict-filter 12,000-SNP reservoir sample generated with seed 20250416 served as an equal-density operational baseline for repeated PCA, accession-ranking, interval, and permutation analyses (Vitter, 1985). Using a fixed marker count provided matched marker density between sgC and sgE despite their different numbers of eligible variants and made repeated sensitivity and permutation calculations directly comparable. The baseline was not used as a substitute for the complete filtered datasets: all eligible variants were included in the streaming PCA and genome-wide differentiation scans. Its adequacy was evaluated against deterministic bottom-k samples of 6,000, 12,000, 24,000, and 48,000 SNPs under five independent seeds (20250416–20250420). Stability was evaluated by pairwise-distance correlations, Procrustes similarity, interval overlap, accession-rank correlations, and top-six membership (**Supplementary Tables S3**, **S4**; **Supplementary Figure S1**).

### 2.4 Population structure and diploid-reference sensitivity

PCA was performed on standardized diploid dosage matrices using five components (Jolliffe and Cadima, 2016). Because the five diploid references are deeply divergent from Arabica and numerically unbalanced, primary inference on within-Arabica structure used the 39-accession Arabica-only panel. The full 44-accession analysis was retained as progenitor context. For each sensitivity condition, Arabica pairwise distances in the two analyses were correlated, and cultivated-introgressed centroid separation was compared (**Supplementary Table S5**; **Supplementary Figure S2**).

### 2.5 Genome-wide differentiation and permutation testing

For the cultivated-versus-introgressed contrast, absolute allele-frequency difference (|ΔAF|) was calculated at every eligible SNP. All-eligible genome-wide summaries were generated for each labelled pseudomolecule and for non-overlapping 1-Mb windows containing at least five valid markers. Chromosomes were ranked by mean |ΔAF|, while operational interval summaries used the locations of the 250 most differentiated markers. Separately, significance was evaluated at the 12,000-SNP baseline marker density by permuting cultivated and introgressed labels 500 times within the strict-filter 12,000-SNP baseline panel while preserving group sizes. The maximum 1-Mb window mean |ΔAF| was recorded for each permutation. Empirical *P* values were calculated as (*b* + 1)/(500 + 1), where *b* is the number of permuted maxima at least as large as the observed baseline-panel maximum (**Supplementary Table S6**; **Supplementary Figure S3**).

### 2.6 Accession prioritization

Accession-level introgression affinity was calculated in the first five Arabica-only principal components. For each accession, Euclidean distance to the cultivated centroid minus distance to the introgressed centroid was used as the affinity score; larger values therefore indicate a position closer to the introgressed than to the cultivated centroid. Introgressed accessions were ranked within each subgenome and sensitivity condition. Robustness was summarized by Spearman rank correlation with the filtered 12,000-SNP baseline and by overlap of the six highest-ranked introgressed accessions (**Supplementary Table S7**).

### 2.7 Direct interval alignment and provisional annotation context

Operational chromosome 4 source intervals from the baseline analysis were extracted from assembly GCA_036785775.1 and divided into 200-kb chunks with 20-kb overlap. Chunks were aligned by NCBI BLAST+ megablast to the corresponding ET-39 sgC and sgE assemblies, retaining high-scoring segment pairs at least 1 kb long and at least 85% identical (Camacho et al., 2009). For each subgenome we quantified source coverage, weighted identity, orientation, source–target order correlation, and the largest alternative-chromosome coverage. This analysis evaluated chromosome-level correspondence and local collinearity; it was not treated as a single exact base-pair liftover.

GFF3 genes overlapping direct alignment-supported target blocks were extracted as the supported gene inventory. Provisional candidates were defined by annotation descriptors associated with defense receptors, kinase signaling, stress response, transcriptional regulation, membrane transport, or leucine-rich repeats. Candidate annotations were not interpreted as causal loci, and exact interval endpoints were not inferred (**Supplementary Tables S8**–**S10**).

### 2.8 Computational environment and reproducibility

Analyses were conducted in Python using NumPy for numerical operations, pandas for tabular processing, and openpyxl for workbook export (McKinney, 2010; Harris et al., 2020). The public repository contains the strict subgenome-filtering, sensitivity, genome-wide differentiation, accession-ranking, direct-alignment, and annotation-export workflows, together with machine-readable source data underlying all figures and supplementary tables.

## 3 Results

### 3.1 Arabica-only population structure after strict subgenome filtering

**Figure 1:**
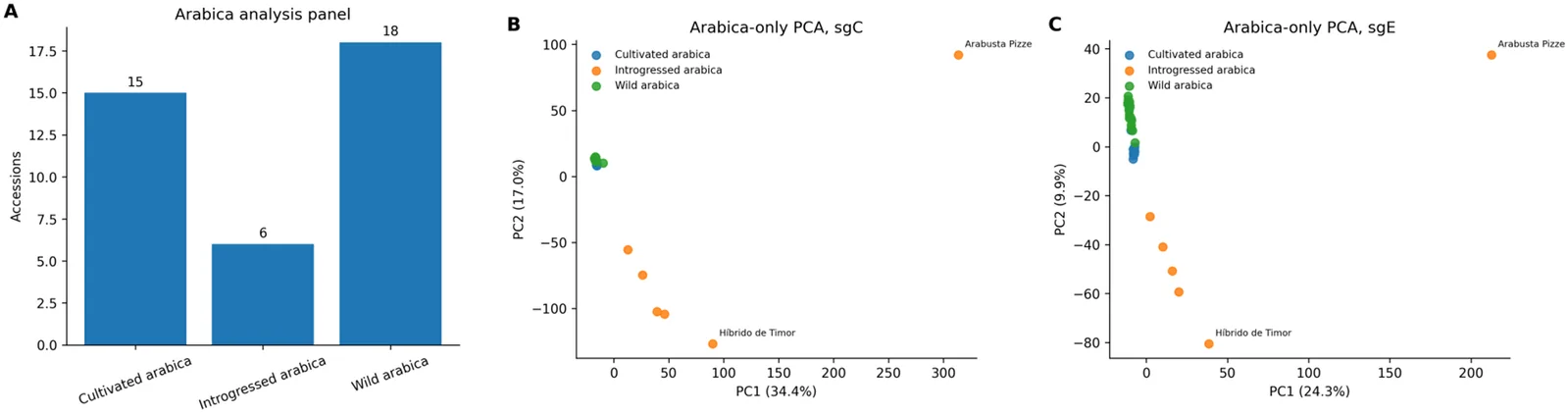
Population structure of the strict subgenome-filtered Arabica panel. **(A)** Composition of the 39-accession *Coffea arabica* analysis panel. **(B, C)** Arabica-only principal component analyses based on the strict sgC- and sgE-labelled pseudomolecule sets, respectively. The five diploid progenitor references are excluded from within-Arabica inference and are retained only in the supplementary full-panel analysis.

After explicit contig-level filtering, both sgC and sgE Arabica-only PCA separated the principal introgressed references from the cultivated and wild accessions (**Figure 1**). Inclusion of the three *C. canephora* and two *C. eugenioides* references changed detailed within-Arabica ordination geometry: the median correlation between full-panel and Arabica-only Arabica distances was 0.860 across sensitivity conditions. However, cultivated-introgressed centroid separation increased in the Arabica-only analysis by approximately 31% in sgC and 83% in sgE. We therefore use Arabica-only PCA for within-Arabica inference and retain the full-panel ordinations only as supplementary progenitor context (**Supplementary Table S5**; **Supplementary Figure S2**).

### 3.2 All-eligible scans support a strong sgC and weaker sgE chromosome 4 signal

**Figure 2:**
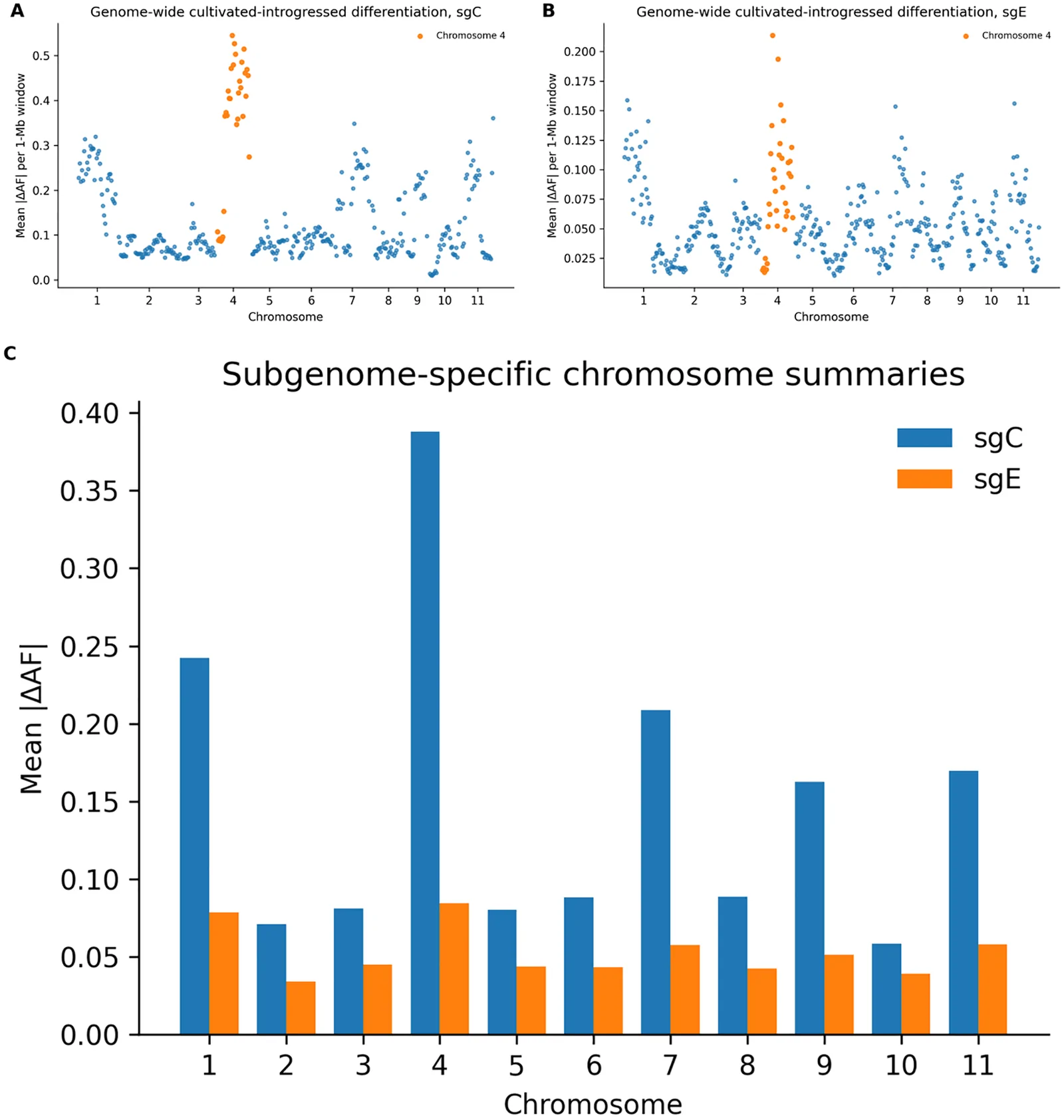
Genome-wide cultivated-introgressed differentiation after strict subgenome filtering. **(A, B)** Mean absolute allele-frequency difference in 1-Mb windows across the 11 sgC- and sgE-labelled pseudomolecules using all eligible variants. Chromosome 4 windows are highlighted. Permutation results for the strict-filter 12,000-SNP baseline panel are shown separately in **Supplementary Figure S3**. **(C)** Chromosome-level mean absolute allele-frequency differences, showing a strong sgC chromosome 4 signal and a weaker chromosome 4-associated signal in sgE.

The all-eligible analysis was genome-wide rather than chromosome 4-targeted. Chromosome 4 was the top-ranked pseudomolecule in both strict subgenome scans (**Figure 2**; **Supplementary Table S6**). In sgC, chromosome 4 had a mean |ΔAF| of 0.3878 and contained 46,552 markers with |ΔAF| at least 0.90; the all-eligible maximum-mean 1-Mb window was 14–15 Mb (mean |ΔAF| = 0.5450). In sgE, chromosome 4 also ranked first but the signal was markedly weaker: mean |ΔAF| was 0.0843, 642 markers had |ΔAF| at least 0.90, and the all-eligible maximum-mean window was 11–12 Mb (mean |ΔAF| = 0.2137). In separate 500-permutation tests of the strict-filter 12,000-SNP baseline, the observed maximum window means were 0.5738 at 14–15 Mb in sgC and 0.2232 at 17–18 Mb in sgE, exceeding null 99th percentiles of 0.3482 and 0.1656, respectively (empirical *P* = 0.001996 and 0.003992). Thus, both subgenomes show chromosome 4-associated differentiation in the same accession contrast, but the magnitude is strongly asymmetric and does not support equivalent introgression.

### 3.3 SNP-panel sensitivity and accession prioritization are reproducible

PCA structure was stable across panel sizes, seeds, and the all-eligible analysis (**Supplementary Table S4**; **Supplementary Figure S1**). The 12,000-SNP density was the smallest tested condition that recovered chromosome 4 as the top-ranked pseudomolecule in all five seeds for both subgenomes. The 6,000-SNP condition was less stable, particularly in sgE, whereas increasing the density to 24,000 or 48,000 SNPs did not materially alter population structure, chromosome ranking, or accession prioritization. Results from the complete filtered variant sets were likewise concordant with the 12,000-SNP baseline: Arabica-only PCA pairwise-distance correlations were 0.9955 for sgC and 0.9996 for sgE, and the same six introgressed accessions remained in the top six under all tested conditions. We therefore retained 12,000 SNPs as the smallest empirically sufficient equal-density baseline rather than as a claim of universal optimality.

**Figure 3:**
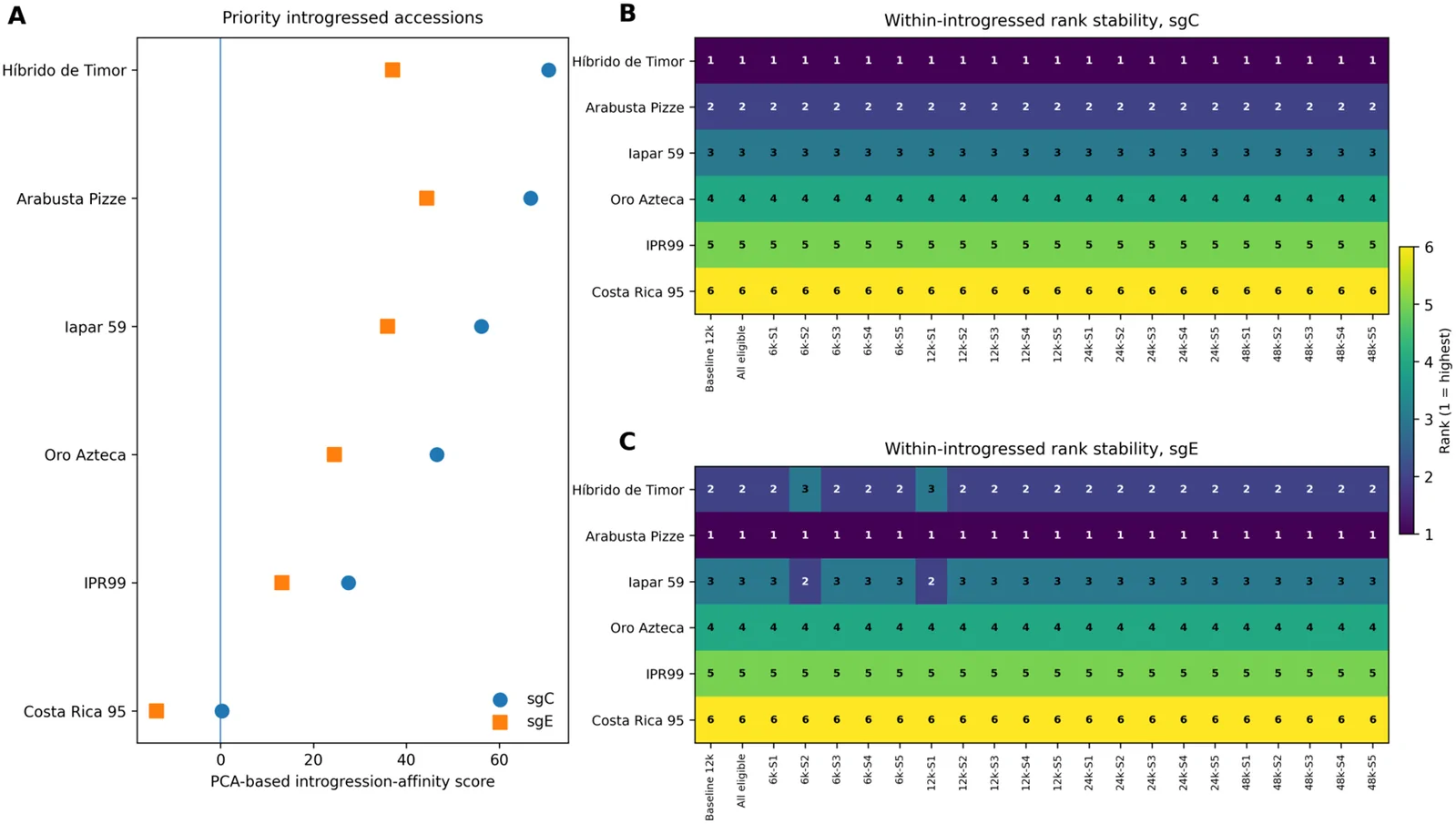
Stability of accession-level prioritization. **(A)** Baseline PCA-based introgression-affinity scores for the six principal introgressed reference accessions. **(B)** Within-introgressed rank stability across SNP-panel sizes, independent seeds, and the all-eligible analysis for sgC. **(C)** Corresponding within-introgressed rank stability for sgE. Rank 1 denotes the highest introgression-affinity score.

Accession prioritization was likewise stable. Median Spearman correlation of accession affinity with the filtered 12,000-SNP baseline was 0.9729, and the same six introgressed references were retained in every condition: Híbrido de Timor, Arabusta Pizze, Iapar 59, Oro Azteca, IPR99, and Costa Rica 95 (**Figure 3**; **Supplementary Table S7**). These accessions include a natural Timor-derived donor, an interspecific Arabusta background, and Catimor- or Sarchimor-derived cultivars distributed through Brazilian and French donor collections. Their geographic source, pedigree context, and published or metadata-based disease and agronomic information are summarized in **Supplementary Table S1**. For example, IPR99 has been reported as resistant to coffee ringspot virus and partially resistant to leaf rust, but no phenotype was measured for any accession in the present study (Sera et al., 2013).

The subgenome-specific affinity profiles further distinguish the principal reference backgrounds. Híbrido de Timor ranked first in sgC under all 22 evaluated conditions (baseline affinity score, 70.66), whereas Arabusta Pizze ranked first in sgE (44.43) and second in sgC. Iapar 59 ranked third in sgC and alternated between second and third in sgE; Oro Azteca, IPR99, and Costa Rica 95 remained fourth, fifth, and sixth, respectively, in both subgenomes. These results identify Híbrido de Timor and Arabusta Pizze as the strongest accession-level anchors of the sgC and sgE components of the cultivated–introgressed contrast, while the four derived cultivars provide stable downstream reference points. The chromosome 4 regions identified by the independent genome-wide scans provide a concrete genomic focus for comparative follow-up among these accessions; however, the present analysis does not infer phased accession-specific introgression boundaries, and the affinity scores do not measure field resistance or agronomic performance.

### 3.4 Direct alignment supports chromosome-level correspondence and a provisional annotation resource

**Figure 4:**
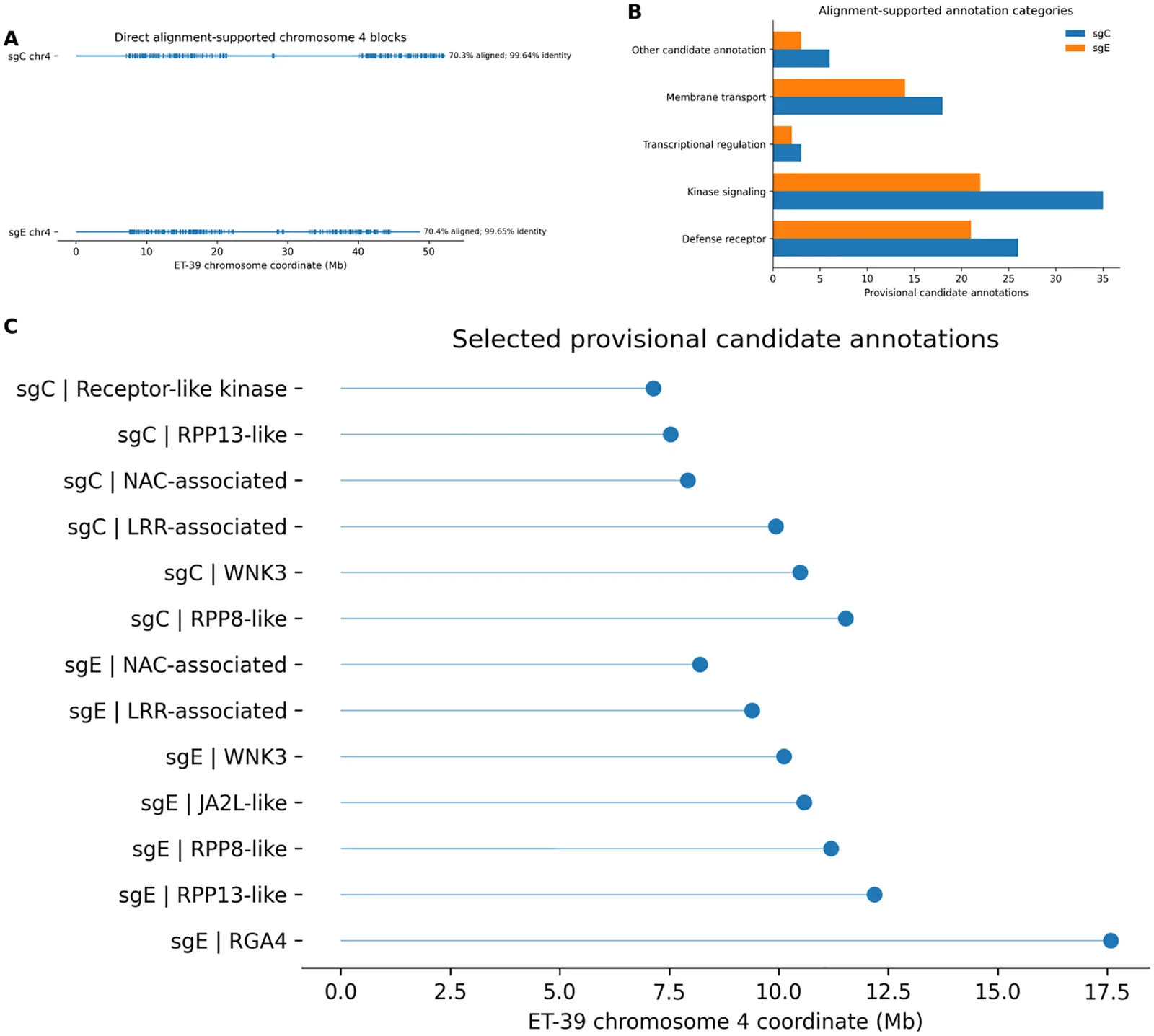
Direct alignment-supported chromosome 4 annotation context. **(A)** Direct sequence-alignment blocks linking the source intervals to the expected ET-39 chromosome 4 pseudomolecules. Percentages report aligned source coverage and weighted nucleotide identity. **(B)** Functional categories among provisional candidate annotations located within alignment-supported target blocks. **(C)** Selected provisional defense-, signaling-, and regulatory-associated annotations. Chromosome-level correspondence is supported, but exact base-pair interval boundaries and causal assignments are not inferred.

Direct sequence alignment assigned all highest-scoring source chunks to the expected ET-39 chromosome 4 pseudomolecules. Retained alignments covered 70.29% of the sgC source interval and 70.39% of the sgE source interval, with weighted identities of 99.643% and 99.653%, respectively (**Figure 4**; **Supplementary Table S10**). Source–target order correlations were 0.9993 in sgC and 0.9999 in sgE, while the largest alternative-chromosome coverage was below 4% in both analyses. These results support chromosome-level correspondence and argue against wholesale relocation to another chromosome. Incomplete coverage and a minority of reverse-orientation blocks nevertheless preclude interpretation as single base-pair-resolved liftover intervals.

Alignment-supported blocks contained 900 sgC and 692 sgE gene models, of which 88 and 62, respectively, met the provisional candidate-annotation criteria (**Supplementary Tables S8**, **S9**). The retained annotations included RPP13-like and RPP8-like resistance proteins, receptor-like kinase and WNK3-associated loci, JA2L-like and other NAC-domain annotations, and leucine-rich-repeat-associated proteins. These features provide biologically plausible annotation context, but they do not establish resistance phenotypes or causal gene function.

## 4 Discussion

This study does not rediscover the Timor-associated chromosome 4 introgression reported by Salojärvi et al. (2024). Its incremental contribution is a quantitative validation layer: strict contig-level subgenome filtering, all-eligible genome-wide scanning, independent permutation testing of the strict-filter 12,000-SNP baseline, multi-panel sampling sensitivity, Arabica-only ordination, stable accession ranking, and direct sequence-alignment-supported annotation. Together, these analyses convert a known biological signal into a reproducible resource whose strongest and weakest claims are explicitly separated.

Explicit subgenome filtering was essential because both public VCFs use a combined Arabica reference and contain sgC-labelled, sgE-labelled, and unassigned contigs. Treating file identity alone as subgenome identity can mix records from the two genomic compartments. The workflow therefore excludes opposite-subgenome and unassigned records before all downstream analyses.

The strict all-eligible genome-wide scan strongly supports the expected sgC result. Chromosome 4 ranked first genome-wide and was recovered in every 12,000-SNP-or-larger replicate; the independent strict-filter 12,000-SNP baseline permutation test also placed its observed maximum above the null distribution. Subgenome-specific breeding signatures have likewise been documented in other allopolyploid crops, where selection can preserve markedly different genomic patterns across homoeologous compartments (Qian et al., 2014; Wang et al., 2022). The sgE chromosome 4 signal also ranked first in the all-eligible scan and was stable at 12,000 SNPs or more, while its baseline-panel permutation maximum exceeded the corresponding null distribution. Its mean effect and number of extreme markers were nevertheless far smaller. We therefore interpret sgE as weaker chromosome 4-associated group differentiation observed in the same accession contrast, not as evidence of direct *C. canephora* introgression, physical linkage to sgC, or a shared causal block. This interpretation is consistent with the source study’s conclusion that recent alien introgression occurred predominantly in sgC.

The accession-ranking output is one of the most reproducible additions. The same six introgressed references were retained across every panel size, seed, and all-eligible analysis. Their backgrounds also make biological sense: Híbrido de Timor anchors the historically important donor lineage; Arabusta Pizze represents a tetraploid Arabica–canephora hybrid background; and Iapar 59, Oro Azteca, IPR99, and Costa Rica 95 represent Timor-derived breeding lineages. Nevertheless, ranking here measures genomic position relative to the cultivated and introgressed centroids, not field performance. The analysis does not establish that a higher affinity score predicts rust resistance, quality, yield, or adaptation.

Direct alignment substantially improves the annotation analysis compared with proportional coordinate scaling. The high nucleotide identities, near-monotonic source–target order, expected chromosome assignments, and low alternative-chromosome coverage support chromosome-level correspondence. At the same time, approximately 30% of each operational source interval lacked retained direct alignment coverage, and some blocks were reversed. Exact boundaries therefore remain unresolved, and the target-coordinate envelopes should not be interpreted as continuous orthologous interval sizes. Restricting the gene inventory to alignment-supported blocks yields a more defensible provisional annotation library, including resistance-protein, receptor-like kinase, NAC, and leucine-rich-repeat classes that are broadly compatible with plant immune signaling (Wu and Zhou, 2013; Stephens et al., 2022; Dong et al., 2024). Coffee genomes contain large NLR repertoires, and comparative resequencing has identified disease-resistance gene families among the most variable genomic compartments across *Coffea* species (Huang et al., 2020; Santos et al., 2022). A recent study also functionally validated a rust race II resistance-associated region in *C. arabica*, illustrating the phenotype-linked follow-up required before candidates from the present analysis can be interpreted functionally (Ariyoshi et al., 2024).

The host-genomic context is relevant to coffee leaf rust, but the present study does not directly test host–pathogen interactions. *Hemileia vastatrix* populations show host adaptation, recombination, and geographic structure (Silva et al., 2018; Rodrigues et al., 2022), and Timor-derived breeding materials have repeatedly been used in rust-resistance improvement. However, no inoculation, transcriptomic, field, or gene-function data were generated here. The accession context and candidate annotations should therefore guide, not substitute for, phenotype-linked validation.

Several limitations remain. First, the study relies on processed public VCFs and inherits their upstream variant-calling choices. Second, although sensitivity analysis demonstrates that the main population structure, chromosome ranking, and accession ranking are stable, operational interval endpoints vary with marker density and should not be interpreted as fixed fine-mapping boundaries. Third, Arabica ordination geometry is influenced by inclusion of the five deeply divergent diploid references; this is why the Arabica-only PCA is used for primary inference. Fourth, direct alignment provides approximately 70% source coverage rather than complete liftover, leaving unresolved sequence and possible local structural differences. Finally, all gene and breeding interpretations remain hypothesis-generating until tested with phenotype, expression, or functional data.

## 5 Conclusion

Strict subgenome filtering, all-eligible genome-wide scans, and independent permutation tests of the strict-filter 12,000-SNP baseline confirm that chromosome 4 is the dominant cultivated–introgressed differentiation signal in sgC and a weaker but significant signal in sgE. The result is stable across SNP panel sizes and seeds, and the six principal introgressed reference accessions are retained under every sensitivity condition. Direct sequence alignment supports chromosome-level correspondence and an alignment-supported provisional annotation resource, but not exact interval boundaries or causal resistance genes. This analysis therefore links the strong sgC and weaker sgE chromosome 4 signals to six consistently prioritized introgressed references. Híbrido de Timor and Arabusta Pizze provide the strongest sgC and sgE affinity anchors, respectively, while Iapar 59, Oro Azteca, IPR99, and Costa Rica 95 provide stable derived breeding references for future chromosome 4 haplotype and phenotype comparisons. These rankings reflect genomic affinity rather than measured field resistance or agronomic performance.

## Data Availability

All datasets analyzed in this study are publicly available. Subgenome-resolved variant calls, accession metadata, syntenic alignments, and documentation are available from Dryad at https://doi.org/10.5061/dryad.qnk98sfpt (Salojärvi, 2023). ET-39 subgenome assemblies and GFF3 annotations are available from Zenodo at https://doi.org/10.5281/zenodo.17250347 (Sotero and Domingues, 2025), and assembly metadata are available through NCBI Datasets under accession GCA_036785775.1 (National Center for Biotechnology Information, 2024). No new sequencing, genotype, phenotype, or experimental data were generated. The analysis workflow for strict subgenome filtering, sensitivity testing, genome-wide differentiation, accession prioritization, direct interval alignment, and annotation export is available at https://github.com/EJSAHN/coffee-arabica-introgression-architecture. Machine-readable source data underlying all figures and supplementary tables are provided with the repository and article.

## Supplementary material

Additional file 1: **Supplementary Tables S1**–**S10**, including the accession context, strict contig-filter audit, validation overview, SNP/PCA sensitivity, diploid-reference sensitivity, separate all-eligible genome-wide and strict-filter 12,000-SNP baseline permutation results, accession-rank stability, alignment-supported gene inventory, provisional candidate annotations, and direct-alignment diagnostics.

Additional file 2: **Supplementary Data S1**, containing the expanded machine-readable outputs underlying the figures, supplementary tables, strict-filter audits, PCA analyses, accession rankings, operational interval summaries, all-eligible genome-wide scans, strict-filter 12,000-SNP baseline permutation tests, direct-alignment blocks, and annotation-supported gene lists.

Additional file 3: **Supplementary Figures S1**–**S3** showing SNP-subsampling stability, diploid-reference sensitivity, and permutation validation of the strict-filter 12,000-SNP baseline panel.
