# Supplementary material for: Genome-wide subgenome-resolved analysis validates chromosome 4 differentiation and prioritizes introgressed Coffea arabica accessions
Source: Front Plant Sci. 2026 Aug 31;17:1925916. doi: 10.3389/fpls.2026.1925916 (PMC13572660; doi:10.3389/fpls.2026.1925916)
Supplement: Supplementary file 1 [file DataSheet1.docx]

**Supplementary Figures**

**Figure S1. SNP-subsampling stability of Arabica-only PCA.**

Pairwise-distance correlations relative to the all-eligible analysis across four SNP panel sizes and five independent seeds for sgC and sgE.

**Figure S2. Effect of diploid reference accessions on Arabica ordination geometry.**

Correlations between Arabica pairwise distances in the full 44-accession panel and the Arabica-only panel across sensitivity conditions. Because the diploid references altered detailed within-Arabica geometry, Arabica-only PCA is used for primary inference.

**Figure S3. Permutation validation using the strict-filter 12,000-SNP baseline panel.**

Null distributions of the maximum 1-Mb window mean absolute allele-frequency difference obtained from 500 group-label permutations of the strict-filter 12,000-SNP baseline panel. Vertical lines indicate the observed baseline-panel maximum for sgC and sgE.
